# Patients with somatoform disorders: More frequent attendance and higher utilization in primary Out-of-Hours care?

**DOI:** 10.1371/journal.pone.0202546

**Published:** 2018-08-30

**Authors:** Ruediger Leutgeb, Sarah Berger, Joachim Szecsenyi, Gunter Laux

**Affiliations:** University Hospital Heidelberg, Department of General Practice and Health Services Research, Heidelberg, Germany; Washington State University, UNITED STATES

## Abstract

**Background:**

One significant health policy challenge in many European countries at present is developing strategies to deal with the increase in patient attendance at Out-of-Hours care (OOHC), whether this is at OOHC-Centres in primary care settings or hospital emergency departments (ED). FAs (FAs) presenting in OOHC are a known challenge and previous studies have shown that FAs present more often with psychological problems and psychiatric comorbidities rather than severe physical complaints. FAs may be also contributing to the rising workload in OOHC-Centres in primary care. The aim of this study was to determine attendance frequencies and health problem presentation patterns for patients with and without somatoform disorders (ICD-10 F45 diagnoses) in OOHC-Centres in primary care. Some of these somatoform disorders may have a psychiatric character. Moreover, we wanted to compare health care utilization patterns (pharmacotherapy and hospitalizations) between these patients groups.

**Methods:**

Routine OOHC data from a large German statutory health insurance company in the federal state of Baden-Wuerttemberg were evaluated. 3,813,398 health insured persons were included in the data set from 2014. The data were initially made available for our study group in order to evaluate a comprehensive evaluation programme in German primary care, the “Hausarztzentrierte Versorgung” (HZV), loosely translated as “family doctor coordinated care”. We used the ICD-10 codes F45.0-F45.9 in regular care to identify patients with somatoform disorders and compared their health care utilization patterns (attendance rates, diagnoses, prescriptions, hospitalization rates) in OOHC to patients without somatoform disorders. Attendance rates were calculated with multivariable regression models in order to adjust for age, gender, comorbidities and for participation in the HZV intervention.

**Results:**

350,528 patients (9.2%) of the 3,813,398 insured persons had an F45-diagnosis. In comparison with the whole study-sample, patients with an F45-diagnosis were on average seven years older (51.7 vs. 44.0 years; p<0,0001) and the percentage of women was significantly higher (70.1% vs 53.3%; p<0,0001). In OOHC, as opposed to normal office hours, the adjusted rate of patients with an F45-diagnosis was 60.6% higher (adjusted for age, gender and co-morbidity) than in the general study-sample. Accordingly, in OOHC, prescriptions for antidepressants, hypnotics, anxiolytics but also opioids were significantly higher than in the general study population i.e. those without F45- diagnoses. However, an F45 diagnosis was only made in 3.45% of all F45 patients seen in OOHC in 2014.

**Conclusions:**

Patients with somatoform disorders were more FAs in both regular office hours and in OOHC in primary care settings. In OOHC, they are normally not identified as such because the somatoform illness is secondary to other acutely presenting symptoms such as pain. While it is acknowledged that it is difficult to make an exact diagnosis in this complex group of somatoform disorders in an OOHC setting, it is still important to develop continuing education programmes for medical staff working in OOHC, to support effective recognition and response to the specific needs of this complex patient group.

## Introduction

In Europe the increasing numbers of patients presenting in an Emergency Department (ED) or in Out-of-Hours care (OOHC) centres [1–4] are causing problems associated with overcrowding, particularly delays in receiving timely medical care. Dealing with the consequences of overcrowding is also an on-going challenge for the doctors on duty [5].

Additionally, a known challenge complicating this issue is the phenomenon of frequent attenders (FAs) presenting in OOHC. There is no standard definition for frequent attendance. In the study of Jacob et al. FAs are defined as attending an ED or OOHC 5 or more times per year. In other studies, moderately FAs are defined as visiting an ED or OOHC between 5–20 times per year, and extremely FAs are defined as visiting an ED or OOHC more than 20 times per year [6,7]. Data from currently available studies on presentation rates for FAs in OOHC is variable. FAs comprise between 7.7% and 20.1% of OOHC patients depending on the setting and country. Sociodemographic characteristics such as social deprivation or low income are factors that have been linked to frequent attendance in OOHC [6,8,9]. Infants and elderly patients are also more likely to be FAs in OOHC. In addition, in some studies, patients with chronic diseases and those suffering from psychiatric disorders, in particular depressions have been identified as frequent or very FAs both in primary care in general and in OOHC care [9–13]. Moreover, FAs with chronic diseases presenting at an OOHC-centre often additionally suffer from major depression. Such additional mental disorders are underreported in OOHC [14].

Patients with somatoform disorders (ICD-10 codes F45.0-F45.9) provide a particular challenge in OOHC care. These disorders are characterized by significant distress or functional impairment associated with one or more somatic symptoms or fear of a serious illness in the absence of somatic symptoms. Diagnosing their (often somatic) complaints for the attending physician can be difficult because of uncertainties involved [15]. Different definitions for similar symptom entities have been developed including: persistent medically unexplained symptoms (MUS), functional somatic symptoms (FSS), bodily distress syndromes (BDS) or somatic symptom disorder (SSD) [16–18]. Previous studies have investigated the diagnoses potentially associated with somatoform disorders in combination with other factors such as treatment options and the health care utilization for all health care problems in this complex patient group as well as their associated health care costs [19,20].

Nevertheless, little published research is available on attendance patterns for patients with somatoform disorders and their health problem presentation patterns in OOHC in primary care settings.

The intention of this retrospective study was to examine medical diagnoses of patients with somatoform disorders based on data from the “Allgemeine Ortskrankenkasse—Baden-Württemberg” (AOK-BW), a large state health insurance company in Germany. Germany wide, 26.29 million people (36.2% of the general population) have insurance by the AOK and in the state of Baden-Wuerttemberg this is currently 4.3 million people. Germany has a policy of universal health coverage. Health insurance is compulsory either through statutory or private health insurance. State health insurance companies provide health cover for 72.76 million people. A further 8.77 million inhabitants with high income (above 59,400 € per year or self-employed persons) are insured by private health insurance companies [21–23].

The aim of this study was to determine attendance frequencies and health problem presentation patterns for patients with and without somatoform disorders in OOHC-Centres in primary care. Moreover, we wanted to compare health care utilization patterns (pharmacotherapy and hospitalizations) between these patients groups.

## Methods

Data for this study were supplied by the AOK state health insurance company Baden-Wuerttemberg, Germany. These data were analyzed cross sectionally in order to determine presentation patterns of patients both with and without somatoform disorders. The eligible study population consisted of 3.81 million insured individuals. The observation period was from 1^st^ January 2014 to 31^st^ December 2014.

The data were initially made available for our study group in order to evaluate a comprehensive evaluation programme in German primary care, the *Hausarztzentrierte Versorgung* (HZV), loosely translated as “family doctor coordinated care”. The HZV is a programme encouraging patients to enroll with a family doctor pursuant to Section 73b, Volume V of the German Social Security Code. It came into effect in Baden-Wuerttemberg on July 1st, 2008. The HZV is aimed at enhancing health care for patients with chronic diseases and complex health care needs e. g., those requiring long term care. This complex intervention, which is voluntary for both family doctors and patients, aims to strengthen the coordination role of family practitioners. As a result, this intervention is believed to increase the quality of medical health care for participating patients and, ideally, to be more cost effective. The details of this intervention are described elsewhere [24,25]. For this study, we did not focus on patients enrolled in the HZV intervention, but were able to use these data to analyse attendance patterns for patients with somatoform disorders and their presentation patterns at OOHC in primary care. OOHC is defined as care during out-of-hours periods where regular medical ambulatory services are not available. In Germany these periods extend from 7:00 p.m. to 7:00 a.m. on Monday, Tuesday and Thursday, and from 2:00 p.m. to 7:00 a.m. on Wednesday and Friday and also on weekends and public holidays. Since introduction of the national emergency number 116117 in 2015 the OOHC periods were adopted in all federal states of Germany. In 2012 about 15% patients walked in an OOHC center without an appointment. New evaluations after the introduction of the national emergency number focusing on that percentage are not yet available [26].

The AOK granted additional permission for the data analysis carried out within this study.

Within the dataset, a specific reimbursement code (particular accounting digits for OOHC services) was used to unambiguously identify health care utilization in OOHC-centres. The ICD-10 codes F45.0-F45.9 [27] were used to identify patients with somatoform disorders. Data on referrals to hospital were also available within the data set. Moreover, due to the central pharmaceutical numbers (“Pharmazentralnummer”, PZN) that can be mapped on the ATC (Anatomical Therapeutic Chemical) classification, we were unambiguously able to determine which medications and active pharmaceutical ingredients were prescribed in OOHC.

Moreover, age and gender was available for every patient within the dataset. Based on the ICD-10, it was possible to determine the Charlson-Index in order to approximate patients’ comorbidity. There are particular diagnoses corresponding to more severe conditions. Values between 1 and 6 are assigned for those diagnoses. Finally, a summary score is determined for each individual. The Charlson Index is a way of adding up chronic conditions and incorporating a severity level. Patients’ Charlson Index was derived based on ICD-10 diagnoses: 17 chronic conditions are weighted by severity, and the weighs of these conditions are added for a summary score of comorbid chronic conditions (range = 0–30). The underlying calculus is described in detail elsewhere [28].

In order to calculate adjusted attendance rates we performed multivariable regression analyses with regard to patients’ age, gender, comorbidity and participation in the HZV intervention (0 = no, 1 = yes). Moreover, we accounted for the hierarchical design of the sample, where patients (level 1) are clustered in OOHC centres (level 2).

Data storage and extraction were performed with MySQL Community Server x64 (Oracle Corporation, Redwood Shores, CA, USA).

In order to calculate frequencies, rates and percentages we used SAS PROC SQL. For the multivariable analyses, we used SAS PROC GENMOD [29] (SAS 9.4 x64, SAS Institute Inc., Cary, NC, USA).

For all analyses, results were considered statistically significant if the p value was 0.05 or less.

Ethical approval for the study was given by the University Hospital Heidelberg Ethics Committee (No. S-359/2013). Patients of the study could not be informed and involved because we obtained pseudonymized data. Since there was no information about patient names, dates of birth or patient residences, the responsible ethics committee had no concerns at all to use these data without informed consent for study purposes.

## Results

**Fig 1:**
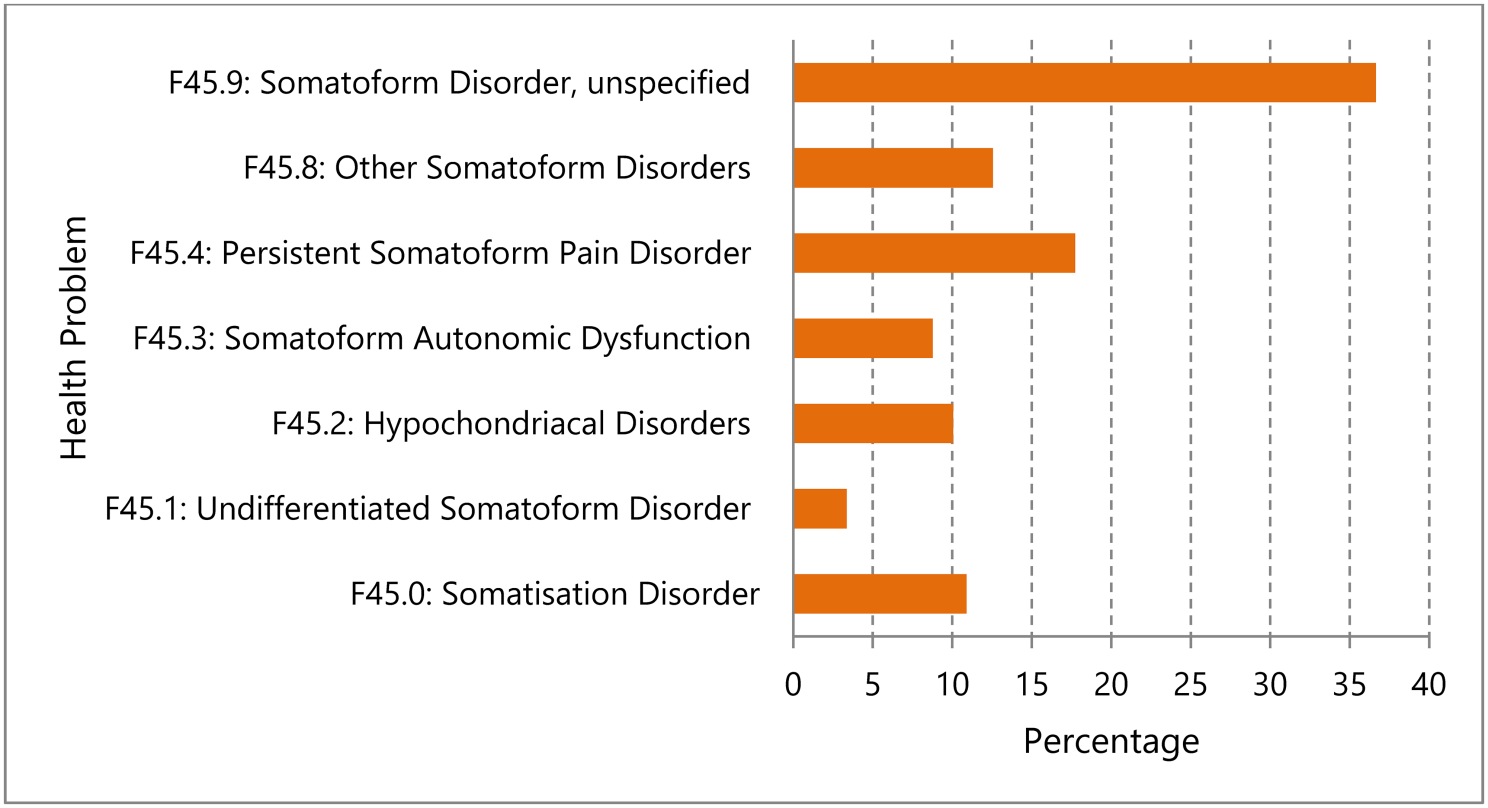
Recorded somatoform disorders F45.

3.81 million insured persons were evaluated in the observation period from 1^st^ January 2014 to 31^st^ December 2014, both during normal office hours and in OOHC in primary care settings. 350,528 (9.2%) of them had a F45-diagnosis of somatoform disorders. Nearly 37% of the F45-diagnoses were coded with “Somatoform Disorder, unspecified”. The distribution of all F45 codes is presented in Fig 1.

Patients with somatoform disorders were on average seven years older and the percentage of female patients was significantly higher in comparison to the study population without somatoform disorders (70.1% vs 53.3%; p<0.0001). The patient characteristics are shown in Table 1.

**Table 1:** Patient characteristics.

	F45-Patients (n = 350,528)	Non-F45-Patients (n = 3,462,870)	P-Value
Age (Mean ± SD)	51.73 ± 19.94	43.26 ± 24.63	<0.0001
Gender male (n, %)	104,737 (29.88%)	1,677,064 (48.43%)	<0.0001
Comorbidity (Mean ± SD)	1.25 ± 1.90	0.80 ± 1.61	<0.0001
HZV Participation (n, %)	125,065 (35.68%)	928,402 (26.81%)	<0.0001
**Adjusted Attendance Rate in OOHC per 100 patients** Mean (Standard Error)	**39.45 (0.0011)**	**24,64 (0.0004)**	**<0.0001**

The adjusted attendance rate for patients suffering from somatoform disorders was 60.1% higher in OOHC than patients without somatoform disorders (contacts per 100 patients per year: 39.5 vs 24.6; p<0.0001). Moreover, the overall comorbidity and being female were positively associated with OOHC contacts, whereas patients’ age and taking part in the GP centred program were not relevantly associated with OOHC contacts. Interestingly, patients with a known F45-diagnosis from regular office hours care that attended an OOHC-centre were identified by the physicians on duty in only 3.45% of cases.

Table 2 shows the relative difference of contacts between patients with an F45-Diagnosis and those without stratified by the number of contacts in OOHC. For patients with five and more contacts the difference was more than 50%.

**Table 2:** Attendance frequencies, relative differences.

	Amount in % (n)		
Contacts in OOHC in 2014	F45-Patients	Non-F45-Patients	F45-Patients: Relative Difference in %
1	70.08 (n = 61,946)	75.74 (n = 476,923)	-8.07
2	19.17 (n = 16,949)	16.96 (n = 106,782)	11.56
3	6.14 (n = 5,425)	4.66 (n = 29,337)	24.09
4	2.32 (n = 2,049)	1.54 (n = 9,686)	33.64
5 or more	2.29 (n = 2,026)	1.11 (n = 6,995)	51.54
Total	100 (n = 88,395)	100 (n = 629,723)	

For patients with an F45 diagnosis, we observed significantly more generalized anxiety (1.10% vs 0.22%) and depressive disorders (0.81% vs 0.26%). An adjusted odds ratio of more than 4.6 for anxiety disorders is considerable. Back pain was the most frequent pain symptom in the general study population but it was significantly more frequent in patients with an F45 diagnosis. Additionally, we found more “other pain symptoms” such as pain in throat and chest, abdominal and pelvic pain and “other soft tissue disorders not elsewhere described” (M79) and not specified by the physicians (see Table 3).

**Table 3:** Comparison of selected health problems.

Health Problem	F45-Patients		Non-F45-Patients		F45- vs. Non-F45-Patients
	Rank	n(%)	Rank	n(%)	Adjusted Odds Ratio 95%-CI; p-value
Back pain	**1**	8,393 (4.99)	**1**	32,634 (3.29)	1.422 [1.387, 1.458]; p<0.0001
Pain localized to upper and lower abdomen pelvic and peritoneal	**2**	7,655 (4.56)	**4**	29,455 (2.97)	1.528 [1.489, 1.569]; p<0.0001
Sore throat and chest pain	**7**	2,656 (1.58)	**21**	9,783 (0.99)	1.671 [1.599, 1,747]; p<0.0001
Other soft tissue disorders	**10**	2,034 (1.21)	**23**	9,501 (0.96)	1.209 [1.152, 1.270]; p<0.0001
Other anxiety disorders	**15**	1,855 (1.10)	**100**	2,160 (0.22)	4.651 [4.363, 4.957]; p<0.0001
Depressive disorders	**26**	1,355 (0.81)	**85**	2,577 (0.26)	2.545 [2.380, 2.720]; p<0.0001

The most frequently prescribed drugs in both patient groups (Non-F45 patients and F45 patients) were anti-inflammatory drugs, analgesics and antipyretics. In keeping with the high frequency of pain symptoms in the F45 patient group, significantly more opioids were prescribed and additionally significantly more prescriptions of antidepressants, sedatives and anxiolytic agents were observed (see Table 4).

**Table 4:** Comparison of frequently prescribed drugs.

Drug Group (ATC 3^rd^ level)	F45-Patients		Non-F45-Patients		F45- vs. Non-F45-Patients
	Rank	n(%)	Rank	n(%)	Adjusted Odds Ratio 95%-CI; p-value
Non-steroidal anti-inflammatory drugs (painkiller for orthopaedic disorders, antirheumatic agents)	**1**	8,117 (10.45)	**1**	68,881 (13.43)	0.967 [0.943, 0.992]; p = 0.0098
Analgesics and antipyretics (painkiller, drugs for fever)	**2**	6,728 (8.66)	**2**	42,225 (8.23)	1.018 [0.991, 1.046]; p: n. s.
Opioids (e.g. morphine)	**5**	3,297 (4.25)	**14**	9,909 (1.93)	1.625 [1.561, 1.693]; p<0.0001
Antidepressants (e.g. Amitriptyline, Citalopram)	**11**	1,824 (2.35)	**25**	4,180 (0.82)	2.014 [1.904, 2.130]; p<0.0001
Hypnotics and Sedatives (Sleep-inducing drugs)	**12**	1,496 (1.93)	**38**	3,224 (0.63)	2.160 [2.029, 2.298]; p<0.0001
Anxiolytics (drugs for anxiety disorders)	**17**	1,236 (1.59)	**40**	3,144 (0.61)	1.892 [1.769, 2.022]; p<0.0001

Finally, we observed an unadjusted frequency of hospitalization for patients with somatoform disorders of 5.73%. The unadjusted frequency of hospitalization for patients without somatoform disorders was 4.99%. However, in our multivariable model we observed that the hospitalization rate for patients with an F45 diagnosis was significantly lower in comparison to Non-F45-patients (OR = 0.972; 95%-CI [0.947; O.997]; p = 0.0262).

## Discussion

In this study sample of 3.81 million AOK Baden-Wuerttemberg state health insured persons, patients with an F45 diagnosis (n = 350,528; 9.2%) had a significantly higher attendance rate in OOHC, as opposed to those without an F45 diagnosis (39.5 vs 24.6 attendances per 100 patients).

These patients had significantly more comorbidities like anxiety or depressive disorders than those patients without an F45 diagnosis. In OOHC, more opioids, antidepressants, hypnotics and sedatives were prescribed for patients with an F45 diagnosis. However, the hospitalization rates of these patients were lower than of the patients without an F45 diagnosis.

In this study, we focussed strictly on somatoform disorders with an ICD-10 code F45.0-F45.9, as it was considered that it would have been confusing to compare all BDS or MUS in regular and OOHC care. The prevalence of F45 diagnoses in this sample (9.2%) was somewhat less than the 16.1%-35.9% prevalence in primary care samples for specific F45 diagnoses (e.g., for BDS [30,31] and MUS [32]), or across F45 diagnoses [33–35]. The lower prevalence of somatoform disorders in this study may be attributable to differences in samples and methodology. For example, our study used insurance data from all patients in a large catchment area who were not systematically screened for F45 diagnoses, compared to volunteering patients who were systematically screened in the other studies [30–35]. The prevalence of other psychiatric diagnoses in OOHC was also lower than those based on systematic evaluation. For example, the WHO estimates the prevalence of depression at 5.2% for Germany, but depression was documented in less than 1% of OOHC patients (see Table 3) [36].

Nearly 37% of patients with an F45 diagnosis in our study were coded “Somatoform disorders, unspecified”. In general, it is difficult to specify somatoform disorders in a primary care setting. GPs would have to read the International classification of diseases (ICD 10) manual of the World Health Organization (WHO) chapter by chapter with the definitions and exclusions to be able to code more precisely [37]. In addition, in day-to-day practice general practitioners make little use of ICD 10 codes representing somatoform health problems [38]. This is in principle a problem of exact coding, to the effect that physicians suppose a specific somatoform health problem but code a more generic diagnosis [39]. Moreover, in OOHC, it is difficult to identify patients with somatoform disorders due to the limited time examining patients and to limited diagnostic equipment such as sonography or blood gas analyzers. These are some of the explanations as to why patients with a known F45-diagnosis from regular office hours care that attended an OOHC-centre were identified by the physicians on duty in only 3.45% of cases.

The consequences of the missing awareness of existing somatoform disorders for this patient group may be a physical diagnosis, in which they may be encouraged to fixate on organic illness symptoms i.e. a “secondary gain” [40]. In the context of OOHC there is no time to address the potential associations between psychosomatic and physical complaints.

Though FAs belong to a heterogeneous population, as previous studies have shown, we could demonstrate that patients with somatoform disorders constitute a core group of FAs [41–43]. The attendance rates of patients with somatoform disorders were 60% higher in OOHC in comparison to patients without somatoform disorders. This finding is in accordance with Jacob et al. [6]. Contrary to their findings, patients in this study were typically seven years older and there was a predisposition to being female [6]. Williams et al. who investigated different subgroups of FAs in OOHC did not see a relation between FAs and higher rates of somatoform disorders. Nevertheless, these results were based on an instrument “SCAN” (a detailed semi-structured psychiatric interview) that was perhaps not sensitive enough to identify patients with somatoform disorders [44,45].

Patients with a known F45-diagnosis in OOHC had significantly more anxiety, depressive episodes and pain disorders as comorbidities and significantly more opioids, antidepressants, and anxiolytics were prescribed by the physicians on duty. Our results show little difference to prescribing patterns in regular office hours for treating such patients [46,47]. It is well known that depression and anxiety, the most common mental disorders, can create and adversely influence comorbidities. Chronic pain is a major issue and the quality of pain is negatively influenced by these mental disorders [48,49]. As our study reveals, there are interconnections between somatoform disorders, depression and anxiety. In OOHC, the potential to implement watchful waiting strategies for patients with somatoform disorders and the mentioned comorbidities, as recommended in primary care, is realistically very limited due to limited consultation times and the inability to follow patients over time [40,50,51].

Jacob et al. have shown that referred patients with somatoform disorders or MUS saw up to five specialists for their symptoms [6]. Patients with somatoform disorders, MUS or BDS have already been associated with increased health care costs for treatment in primary care during regular office hours [52]. The implications for escalating health care costs for this patient group due to their frequent attendance in OOHC adds another reason as to why it is important to accurately diagnose patients with somatoform disorders. The identification of patients with somatoform disorders is over and above the need to ensure effective treatment and reduce potential iatrogenic harm [46] and could be seen as quaternary prevention in best practice primary care [51,53].

Jelinek et al. observed FAs as a population more often self-referred with psychosocial problems but with lower acuity of complaints and therefore lower hospitalization rates. In contrast, moderately FAs present with symptoms of higher urgency and have higher hospitalisation rates [7]. Looking at our results, the subgroup of FAs with a significantly higher contact frequency in OOHC could be considered as belonging to a population with high psychological strain and the sense of being taken care of but not with the requirement of being acutely hospitalized.

### Strengths and limitations of the study

To our knowledge, this is the first study examining attendance patterns for patients with somatoform disorders in OOHC in primary care settings in Germany.

One of the strengths of this study is the comprehensive sample of data covering one whole federal state of Germany both in rural and urban areas with diagnoses in primary care.

In terms of limitations, we know that the rate of patients with MUS or BDS is much higher than the patient sub-group we examined and we do not know if physicians coded the difficult and time-consuming complaints of patients with somatoform disorders accurately. However, this is a general problem of coding and the revision of the ICD-11 classification will perhaps lead to more clarity concerning these challenging differential diagnoses [54]. The next version of the ICD may include BDS, which would provide more diagnostic specificity [52].

Another limitation, given the German setting of this study, relates to referrals to specialists in ambulatory health care as they are not documented in OOHC. So, this further possibility to estimate if patients with an F45 diagnosis were referred on to medical specialists could not be included in this study.

A final limitation in this study is the limited sociodemographic data available within this routine data set. Patients’ subjective assessments of their diseases and sociodemographic information such as education level, income level, disability status, socio-economic status are all relevant factors when considering FAs in OOHC.

Buja et al. showed that low income and disability were associated with an FA status [8]. Worry, being seriously ill, socio-economic problems like unemployment, social deprivation and family dysfunction could increase the psychological strain of somatic complaints and could lead to seeking professional help in OOHC, particularly at nights or on weekends when their own GP was not available [8, 55, 56]. These factors could not be taken into account in our quantitative study.

#### Implications for Clinical practice and future research

It would be of great value to identify patients with somatoform disorders in order to improve their care and treatment, especially in terms of prescriptions, referrals to medical specialists and in some cases hospitalizations.

In the future, we have to better address the needs of patients with unexplained somatic symptoms in OOHC. A standardization of terms describing bodily symptoms and psychological components to these primarily physically perceived symptoms could be particularly helpful to identify these patients. In this context the terms FSS and BDS are increasingly favored to define symptoms and behaviors of this patient group. The primary health care section of ICD-11 will likely include a BDS category in the new revision [57,58].

Implementation of training programs for how to manage this subgroup of patients with somatoform disorders could possibly prevent overtreatment and perhaps reduce the attendance frequency in OOHC [59].

The problem of the availability of patient related information between different health care providers remains an ubiquitous problem in the German health care setting [60].

In further research steps, integrating the patient perspective on their needs and wishes is a further element in the drive to achieving best practice in OOHC.

## Conclusions

Patients with diagnoses of F45 in primary care are FAs in OOHC. The attendance frequency of these patients was 60% above the rate of patients without such a diagnosis. F45 patients were over 50% more likely to make 5 or more OOHC visits in one year than were patients without F45 diagnoses. F45 patients were mostly not identified in the setting of OOHC. Depressive episodes, anxiety and pain disorders were more prevalent for F45-Patients who obtained significantly more anxiolytics, antidepressants and opioids in OOHC. However, the hospitalization rate of these patients was lower consistent with lesser medical acuity of their complaints.

Further research endeavors should be done in order to assess if the psychosomatic treatment services in regular care and OOHC are sufficient for increasing numbers [61] of patients with somatoform disorders.

## Supporting information

S1 Table Supplement 1. Study Data.
(ZIP)

## Abbreviations

AOK-BW: Allgemeine Ortskrankenkasse Baden-Württemberg
ATC: Anatomical Therapeutic Chemical Classification
BDS: Bodily Distress Syndrome
ED: Emergency Department
FAs: FAs
FSS: Functional Somatic symptoms
HZV: Hausarztzentrierte Versorgung
ICD-10: International Statistical Classification of Diseases and related health problems 10th Revision
MUS: Medically Unexplained Symptoms
OOHC: Out-of-Hours Care
SSD: Somatic Symptom Disorder
WHO: World Health Organisation
